# Enhancement of Behavioral Sensitization, Anxiety-Like Behavior, and Hippocampal and Frontal Cortical CREB Levels Following Cocaine Abstinence in Mice Exposed to Cocaine during Adolescence

**DOI:** 10.1371/journal.pone.0078317

**Published:** 2013-10-21

**Authors:** Maria Cristina Valzachi, Elizabeth Teodorov, Tania Marcourakis, Alexis Bailey, Rosana Camarini

**Affiliations:** 1 Departamento de Farmacologia, Instituto de Ciências Biomédicas, Universidade de São Paulo, São Paulo, SP, Brazil; 2 Centro de Matemática, Computação e Cognição, Universidade Federal do ABC, São Paulo, SP, Brazil; 3 Departamento de Análises Toxicológicas e Clínicas, Faculdade de Ciências Farmacêuticas, Universidade de São Paulo, São Paulo, SP, Brazil; 4 Department of Biochemistry & Physiology, Faculty of Health & Medical Sciences, University of Surrey, Guildford, Surrey, United Kingdom; Nathan Kline Institute for Psychiatric Research and New York School of Medicine, United States of America

## Abstract

Adolescence has been linked to greater risk-taking and novelty-seeking behavior and a higher prevalence of drug abuse and risk of relapse. Decreases in cyclic adenosine monophosphate response element binding protein (CREB) and phosphorylated CREB (pCREB) have been reported after repeated cocaine administration in animal models. We compared the behavioral effects of cocaine and abstinence in adolescent and adult mice and investigated possible age-related differences in CREB and pCREB levels. Adolescent and adult male Swiss mice received one daily injection of saline or cocaine (10 mg/kg, i.p.) for 8 days. On day 9, the mice received a saline injection to evaluate possible environmental conditioning. After 9 days of withdrawal, the mice were tested in the elevated plus maze to evaluate anxiety-like behavior. Twelve days after the last saline/cocaine injection, the mice received a challenge injection of either cocaine or saline, and locomotor activity was assessed. One hour after the last injection, the brains were extracted, and CREB and pCREB levels were evaluated using Western blot in the prefrontal cortex (PFC) and hippocampus. The cocaine-pretreated mice during adolescence exhibited a greater magnitude of the expression of behavioral sensitization and greater cocaine withdrawal-induced anxiety-like behavior compared with the control group. Significant increases in CREB levels in the PFC and hippocampus and pCREB in the hippocampus were observed in cocaine-abstinent animals compared with the animals treated with cocaine in adulthood. Interestingly, significant negative correlations were observed between cocaine sensitization and CREB levels in both regions. These results suggest that the behavioral and neurochemical consequences of psychoactive substances in a still-developing nervous system can be more severe than in an already mature nervous system.

## Introduction

 Several behavioral and neurochemical differences exist between adolescence and adulthood. Higher risk-taking and novelty-seeking behavior is a common characteristic of the adolescent period of life [1], which may lead to an increased risk and prevalence of drug abuse. The conditioned place preference (CPP) model of relapse has shown that adolescent rats exhibit delayed extinction and a stronger preference for cocaine-paired cues following priming injections of cocaine in a CPP reinstatement protocol, suggesting a higher risk of relapse in adolescents compared with adults [2].

 Profound developmental changes occur during adolescence. The higher susceptibility to drug addiction in adolescents is partially linked to an immature prefrontal cortex (PFC). The PFC plays an essential role in goal-directed behavior, executive function, and attention performance [3,4]. In humans, the PFC presents delayed development compared with other cortical areas, indicated by synaptic pruning and a decrease in axonal myelination in gray matter throughout adolescence [5,6]. These alterations likely affect characteristic adolescent behaviors, such as the control of impulsivity and decision making, placing adolescents at a greater risk of developing substance use disorders [7].

 Another region that undergoes developmental changes during adolescence is the hippocampus. The hippocampus is especially involved in memory and learning processes and plays an important role in aberrant learning in response to drug addiction-related behavior [8]. These brain areas reach maturity later than primary sensory-motor brain areas [9], which limits motivational inhibitory capacity during early developmental periods. For example, hippocampal glucocorticoid receptors undergo severe pruning during adolescence before maturation in adulthood [10]. This is generally thought to underlie adolescents’ increased vulnerability to stress [11], which may lead to an increased risk of substance use disorders.

 Behavioral sensitization is characterized by an enhanced locomotor response following repeated exposure to psychostimulants and other drugs of abuse [12] and is associated with the incentive salience of a drug. The PFC has been shown to play a key role in the development of psychostimulant-induced behavioral sensitization. Sensitized responses to cocaine have been associated with reduced dopaminergic transmission in the PFC [13]. The direct connection between the PFC and nucleus accumbens, a region involved in motivation and behavioral sensitization, via glutamatergic projections further highlights the key involvement of the PFC in the development of behavioral sensitization.

 A large body of evidence demonstrates the possible molecular mechanisms that underlie behavioral sensitization to psychostimulants [14,15]. Changes in transcription factors and gene expression contribute to persistent alterations in addictive-related behaviors that occur especially in motivational circuitry. Particularly interesting is the involvement of cyclic adenosine monophosphate (cAMP) response element binding protein (CREB) in this phenomenon. Alterations in ΔFosB, CREB, and phosphorylated CREB (pCREB) have been reported after repeated cocaine administration, mainly in the nucleus accumbens and striatum in adult rodents [16,17]. Fasano et al. [17] reported that the inhibition of striatal CREB enhanced locomotor sensitization to cocaine in adult mice. Consistent with this study, Walters and Blendy [18] demonstrated that transgenic mice that overexpressed a mutant form of CREB that had reduced transcriptional activity exhibited increased cocaine-induced locomotor activity compared with wildtype mice. These studies suggest a key modulatory role for CREB in behavioral sensitization to cocaine in adults. Changes in the phosphorylation and activity of CREB have been observed in the PFC and hippocampus following the use of cocaine and other addictive drugs in adult rodents [19,20]. However, the role of CREB in the modulation of behavioral sensitization to cocaine in mice exposed to the drug during adolescence has not yet been demonstrated.

 Acute and chronic cocaine administration and subsequent withdrawal can cause anxiety-like symptoms in rats and mice [21,22]. The emergence of this anxiety-like behavior is considered clinically relevant because it may serve as a motivational trigger for relapse [23]. Unclear is whether the PFC is involved in the development of this anxiety-like behavior in response to cocaine and cocaine withdrawal. Nonetheless, ethanol withdrawal-related anxiety has been associated with decreased CRE DNA binding activity in the rat cortex [24], suggesting that neuroadaptations in cortical CREB may also be involved in the manifestation of anxiety-like behavior following cocaine withdrawal.

 We hypothesized that CREB and pCREB are differentially regulated in the PFC and hippocampus, depending on whether cocaine exposure occurs during adolescence or adulthood. We also evaluated the possible relationship between behavioral responses to cocaine administration and cocaine-regulated CREB and pCREB expression. The present study confirmed our previous findings that adolescents are more sensitive to cocaine-induced behavioral sensitization than adult mice. We further demonstrated a negative correlation between sensitization and CREB and pCREB in the PFC and pCREB in the hippocampus in mice exposed to cocaine during adolescence but not in adulthood. These results shed further light on the molecular mechanisms that underlie the behavioral consequences of cocaine abuse during adolescence, which may explain the higher risk of vulnerability to addiction during this developmental period of life.

## Materials and Methods

### Animals

All of the procedures were approved by the Ethical Committee for Animal Use of the Instituto de Ciências Biomédicas, Universidade de São Paulo (Permit no. 032-126/02). Male adolescent Swiss-Webster mice (postnatal day [PND] 28-30 at the start of the experiments) and male adult mice (PND65-70 at the start of the experiments) were obtained from the Instituto de Ciências Biomédicas, Universidade de São Paulo (São Paulo, Brazil). The animals were housed five per cage in standard polycarbonate cages under controlled temperature (21 ± 1°C) and a 12 h/12 h light/dark cycle with lights on at 7:00 AM. The mice were allowed to acclimate to the housing conditions for 7 days before the initiation of the experiments and had free access to food and water. A total of 98 mice were used. The site of the injections was alternated, and all efforts were made to minimize suffering.

### Drugs and antibodies

 Cocaine hydrochloride (Merck AG, Darmstadt, Germany) was dissolved in physiological saline (0.9% w/v sodium chloride) and administered intraperitoneally (i.p.) in a volume of 1 ml/100 g body weight at a dose of 10 mg/kg. The antibody for Ser133 pCREB was obtained from Millipore (Billerica, MA, USA). CREB was obtained from Santa Cruz Biotechnology (Santa Cruz, CA, USA), and their secondary anti-rabbit antibodies were obtained from Jackson Immuno Research Laboratories (West Grove, PA, USA). The antibody for α-tubulin and its secondary anti-mouse antibody were obtained from Sigma-Aldrich (St. Louis, MO, USA).

### Open-field test

 All of the behavioral procedures were performed during the light phase of the light/dark cycle between 10:00 AM and 2:00 PM. Two days before starting the experiments (H1 and H2), the mice received a daily saline injection and were subjected to the open-field test (40 cm diameter arena, surrounded by a 50 cm wall) to minimize stress induced by experimenter handling, the injection procedures, and exposure to the novel environment. For the next 8 consecutive days (D1 to D8), half of the mice were injected with saline, and the other half were injected with cocaine (10 mg/kg, i.p). On Day 9 (D9), every mouse received a saline injection to evaluate a possible contextual conditioning effect. From Day 10 to Day 19 (D10 to D19), the animals received no injections (abstinence period). On the challenge day (D20), each group was subdivided into two subgroups. Half of animals received a saline challenge injection, and the other half received a cocaine challenge injection, constituting the control group (daily saline injection from D1 to D8 and saline challenge on D20), acute cocaine group (daily saline injection and cocaine challenge), abstinence group (daily cocaine injection and saline challenge), and repeated cocaine group (daily cocaine injection and cocaine challenge). Total locomotor activity was measured for 15 min in the open-field test immediately following the injection on Days 1, 8, 9, and 20 (Table 1). On the other days, the mice received the injections in the home cage. Rodents are well known to exhibit greater peripheral locomotion close to the walls of the open field and avoid the central area, an effect referred to as thigmotaxis. Therefore, the central locomotion/total locomotion ratio was used as an index of anxiety-like behavior [25,26]. Importantly, the data shown on Day 20 were recorded when the adolescent group reached the postpuberty/young adult phase. The timeline in Table 1 depicts the ages at which the mice went through each step of the experimental design. Each trial was recorded by a digital camera, and the arenas were cleaned after each test with 5% ethanol. EthoVision 3.1 software (Noldus Information Technology, Leesburg, VA, USA) was used to track and quantify the total and central distance traveled by each animal. This protocol was adapted from Camarini et al. [27].

**Table 1:** Experimental design.

**Initial Group**	**H1**	**H2**	**D1**	**D2-7**	**D8**	**D9**	**D10-17**	**D18**	**D19**	**D20**	**Final Group**
**Saline**	**Sal (OF)**	**Sal (OF)**	**Sal (OF)**	**Sal (HC)**	**Sal (OF)**	**Sal (OF)**	**N (HC)**	**N (EPM)**	**N (HC)**	**Sal (OF)**	**Control**
	**Sal (OF)**	**Sal (OF)**	**Sal (OF)**	**Sal (HC)**	**Sal (OF)**	**Sal (OF)**	**N (HC)**	**N (EPM)**	**N (HC)**	**Coc (OF)**	**Acute**
**Cocaine**	**Sal (OF)**	**Sal (OF)**	**Coc (OF)**	**Coc (HC)**	**Coc (OF)**	**Sal (OF)**	**N (HC)**	**N (EPM)**	**N (HC)**	**Coc (OF)**	**Repeated**
	**Sal (OF)**	**Sal (OF)**	**Coc (OF)**	**Coc (HC)**	**Coc (OF)**	**Sal (OF)**	**N (HC)**	**N (EPM)**	**N (HC)**	**Sal (OF)**	**Abstinence**
**PND**	**28**	**29**	**30**	**31-36**	**37**	**38**	**39-46**	**47**	**48**	**49**	**Adolescents**
	**65**	**66**	**67**	**68-73**	**74**	**75**	**76-83**	**84**	**85**	**86**	**Adults**

PND, postnatal day; H1 and H2, habituation (the mice received a daily saline injection and were exposed to the open-field test [OF]). D1 to D8, repeated treatment (half of the mice received a daily saline injection [initial Saline group], and the other half received a daily cocaine injection [initial cocaine group; 10 mg/kg, i.p.] in the home cage [HC], except on D1 and D8, when the animals were exposed to the OF. D9, contextual conditioning effect (every mouse received a saline injection and was exposed to the OF. D10 to D19, abstinence period (mice received no injections [N] and were tested in the elevated plus maze [EPM] on D18). D20, challenge day (half of the mice received a saline challenge injection, and the other half received a cocaine challenge injection, constituting the final groups: control, acute, repeated, and abstinence). The animals were immediately exposed to the OF and euthanized 1 h after the last injection.

### Elevated plus maze

During the withdrawal period on Day 18, the mice were evaluated in the elevated plus maze (EPM). The apparatus consisted of two open arms (33.5 × 7.0 cm) and two enclosed arms (33.5 × 7.0 × 19.0 cm) arranged such that the two arms of each type were perpendicular to each other. The maze was elevated to a height of 50 cm. The measures were recorded over a 5 min test period by a blind observer sitting approximately 1 m from the apparatus. The values obtained were converted into percentages of the time spent on the open arms relative to the total time spent in both arms. The number of entries into the open arms was also assessed. An entry into an arm was considered valid only when the animal’s four paws were inside the arm. The EPM was cleaned after each test with 5% ethanol.

### Protein extraction

 One hour after the challenge injection on Day 20, the mice were euthanized by cervical dislocation. The PFC and hippocampus were dissected, immediately placed on dry ice, and stored at -80°C. Nuclear extracts were prepared as described by Rong and Baudry [28] with some modifications. Briefly, the tissues were homogenized in 280-300 μl lysis buffer (10 mM HEPES, 1.5 mM MgCl_2_, 10 mM KCl, 0.1 mM ethylenediaminetetraacetic acid [EDTA], 0.5 mM dithiothreitol [DTT], 0.5 mM phenylmethylsulfonyl fluoride [PMSF], 2 μg/ml leupeptin, 2 μg/ml antipain, 10 mM sodium β-glycerophosphate, 50 mM NaF, and deionized water) and incubated on ice for 10 min. Afterward, 7-7.5 μl of a 10% NP-40 solution was added, vigorously mixed, and centrifuged for 1 min at 16,000 × *g* at 4°C. Pelleted nuclei were resuspended in 60-70 μl extraction buffer (20 mM HEPES, 25% glycerol, 1.5 mM MgCl_2_, 300 mM NaCl, 0.25 mM EDTA, 0.5 mM DTT, 0.5 mM PMSF, 2 μg/ml leupeptin, 2 μg/ml antipain, 10 mM sodium β-glycerophosphate, 50 mM NaF, and deionized water), incubated on ice for 20 min, and then centrifuged for 20 min at 16,000 × *g* at 4°C. The supernatants that contained the nuclear proteins were stored at -80°C. The protein concentration was determined using the Bradford method [29].

### Western immunoblotting

 The extracts (10 μg protein) were loaded on 10% sodium dodecyl sulfate polyacrylamide gel at 100 V for 1 h, transferred to a 0.45 μm nitrocellulose membrane (Bio-Rad Laboratories, Hercules, CA, USA) at 100 V for 1 h, and blocked in blocking buffer (5% non-fat dried milk in 100 mM Tris-base [pH 8.0], 150 mM NaCl, and 0.05% Tween 20) for 1 h. Membranes were probed with a primary antibody to Ser133 pCREB (1:750) or CREB (1:1000) overnight at 4°C or α-tubulin (1:10000) for 1 h at 22.5°C. The membranes were then washed twice for 10 min each in TTBS (100 mM Tris-base [pH 8.0], 150 mM NaCl, and 0.05% Tween 20) and exposed to horseradish peroxidase-linked anti-rabbit (1:50000 for pCREB and CREB) or anti-mouse (1:6000 for α-tubulin) antibody for 45 min at 22.5°C. The blots were washed twice for 10 min in TTBS and once for 10 min in TBS (100 mM Tris-base [pH 8.0] and 150 mM NaCl). Protein bands were detected by ECL plus and exposed on ECL Hyperfilm (GE Healthcare Bio-Sciences, Little Shalfont, Buckinghamshire, UK). The films were scanned, and the optical density was determined using ImageJ software (National Institutes of Health, Bethesda, MD, USA).

 For the Western blot data analysis, the optical density of the bands for the control group was averaged for each gel, and the optical densities of the bands for the cocaine-treated groups were calculated as a percentage of the control value for that gel. Individual pCREB values were divided by their respective CREB values to obtain pCREB/CREB ratio values.

### Statistical analysis

 Statistica 7.0 software (Statsoft, Tulsa, OK, USA) was used for all of the statistical analyses. Analysis of variance (ANOVA) was used to evaluate all of the data, followed by the Newman-Keuls *post hoc* test. Two-way repeated-measures ANOVA (age × days) was used to analyze the habituation effect between the first days after the saline injections. A 2 (age: adolescent vs. adult) × 2 (pretreatment: saline vs. cocaine) × 2 (day: D1 vs. D8) factorial design was used to analyze the development of behavioral sensitization. The analysis of the Pavlovian conditioning of the cocaine-induced locomotor response to a saline injection on Day 9 was conducted using a 2 (age: adolescent vs. adults) × 2 (pretreatment: saline vs. cocaine) design. A 2 (age: adolescent vs. adult) × 2 (pretreatment: saline vs. cocaine) × 2 (challenge injection on Day 20: saline vs. cocaine) factorial design was used to analyze the expression of behavioral sensitization. Thigmotaxis was analyzed after one cocaine injection (basal anxiety levels) and after repeated cocaine injections using a 2 (age: adolescent vs. adult) × 2 (pretreatment: saline vs. cocaine) × 2 (day: D1 vs. D8) design. In all of the experiments, “day” was the within-group factor. We used a 2 (age: adolescent vs. adult) × 2 (pretreatment: saline vs. cocaine) two-way ANOVA to evaluate anxiety-like behavior on the elevated plus maze. A 2 (age: adolescent vs. adult) × 2 (pretreatment: saline vs. cocaine) × 2 (challenge injection on Day 20: saline vs. cocaine) factorial design was used to analyze the CREB and pCREB data individually and pCREB/CREB ratios. When necessary, the ANOVAs were deconstructed.

 Correlations were performed to assess the relationships between the locomotor-stimulatory effects of repeated cocaine administration (i.e., sensitization effect) and CREB and pCREB levels in the hippocampus and PFC for both ages on the test day (Day 20) using the Pearson correlation test. The data are expressed as mean ± SEM. The level of significance was set at 0.05.

## Results

### Total locomotor activity

**Figure 1:**
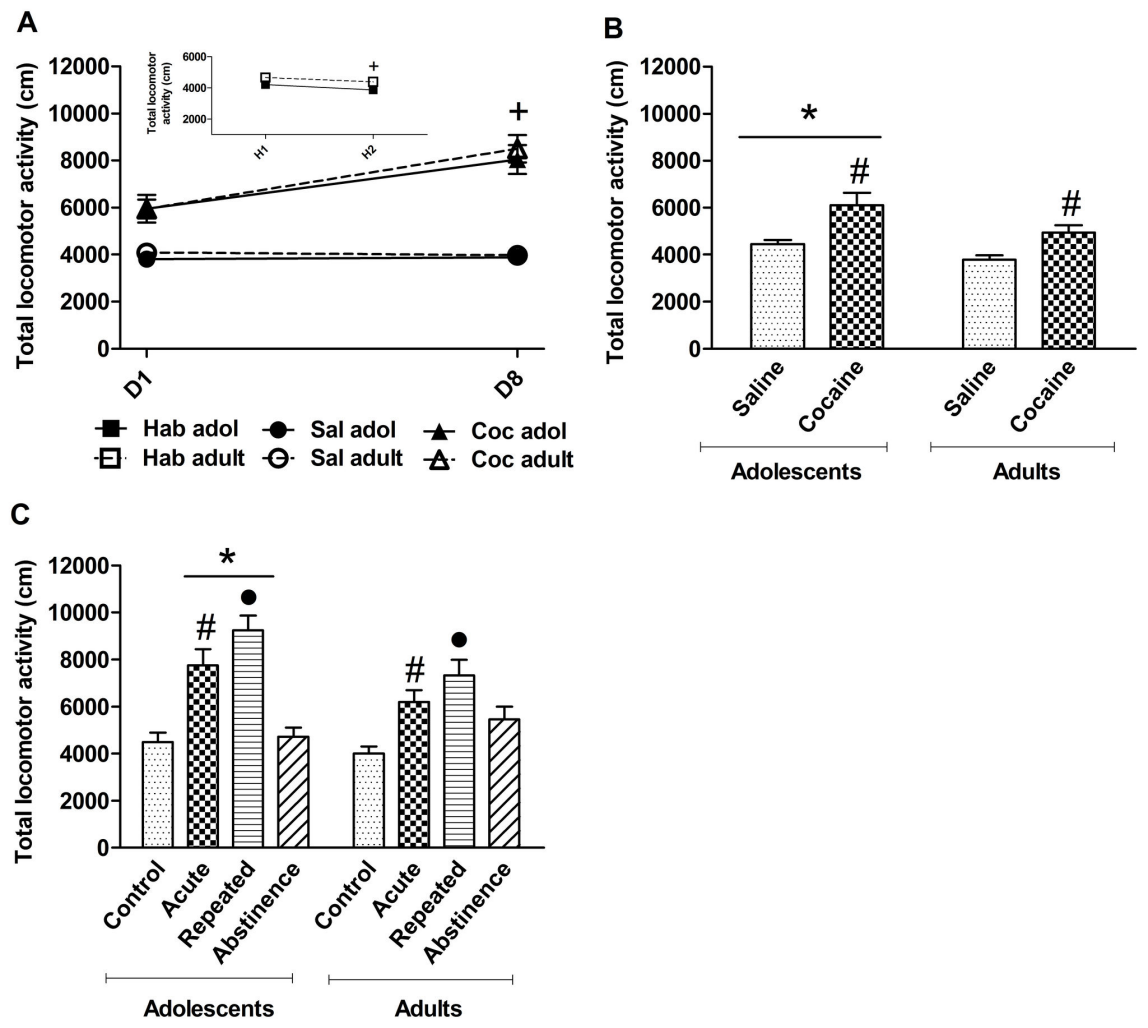
Total locomotor activity in the open field test. (**A**) Total locomotor activity on the habituation days and during repeated saline or cocaine injection. Total locomotor activity in mice on H2 was lower than on H1 in both age groups (^+^ *p* < 0.01, *vs*. H1; *n* = 50 mice, adolescent group; *n* = 48 mice, adult group). Both adolescent and adult mice treated with cocaine exhibited greater locomotor activity on D8 than on D1 (^+^ *p* < 0.01, *vs*. D1; *n* = 25, saline adolescent [Sal adol]; *n* = 25, cocaine adolescent [Coc adol], acute cocaine; *n* = 23, saline adult [Sal adult]; *n* = 25, cocaine adult [Coc adult]). (**B**) Effects of saline injection on total locomotor activity on D9 after repeated injections of saline or cocaine. Animals pretreated with cocaine exhibited greater locomotor activity than animals pretreated with saline (^#^ *p* < 0.01, *vs*. saline). Adolescents exhibited greater increases in activity than adult mice (*p < 0.05, *vs*. adults). (**C**) Effect of a challenge injection of saline or cocaine on total locomotor activity on D20. Acute and repeated cocaine induced more locomotor activity in adolescents than in adults (*p < 0.05, *vs*. adults, age × challenge injection interaction). Mice treated with acute cocaine exhibited greater locomotor activity compared with the control groups, regardless of age (^#^ *p* < 0.01, *vs*. control, pretreatment × challenge injection interaction). Repeated cocaine administration produced greater locomotor activity compared with the acute cocaine group, regardless of age (^●^ *p* < 0.05, *vs*. acute, pretreatment × challenge injection interaction). Adolescent groups: control (*n* = 12), acute cocaine (*n* = 13), abstinence (*n* = 11), repeated cocaine (*n* = 14). Adult groups: control (*n* = 11), acute cocaine (*n* = 12), abstinence (*n* = 12), repeated cocaine (*n* = 13). The data are expressed as mean ± SEM.

Two days before the start of the experiments, the mice received a daily saline injection and were exposed to the open-field test (H1 and H2; Figure 1A). The two-way repeated-measures (day) ANOVA (age × day) revealed a significant effect of day (*F*_1,96_ = 14.4, *p* < 0.01), with a decrease in total locomotor activity at H2 compared with H1 (habituation effect; Figure 1A).

Figure 1A also shows total locomotor activity on the first and eighth days following the daily injections of cocaine or saline in adolescent and adult mice. The three-way repeated-measures (day) ANOVA (age × pretreatment × days) revealed a pretreatment effect (*F*_1,94_ = 68.04, *p* < 0.01), indicating greater total locomotor activity in cocaine-treated mice compared with saline-treated animals. A day effect (*F*_1,94_ = 41.53, *p* < 0.01) and pretreatment × day interaction (*F*_1,94_ = 43.56, *p* < 0.01) were also observed. The *post hoc* analysis revealed an increase in total locomotor activity in cocaine-treated mice on D8 compared with D1, indicating the development of locomotor sensitization at both ages. No effect of age was found for any of the parameters analyzed (*p* > 0.05), indicating a lack of behavioral differences between adults and adolescents in these parameters.

To test the conditioning effect of cocaine, the mice were tested in the open field after a saline injection on Day 9 of treatment (Figure 1B). The two-way ANOVA (age × pretreatment) revealed effects of age (*F*_1,94_ = 10.54, *p* < 0.01) and pretreatment (*F*_1,94_ = 24.33, *p* < 0.01) but no interaction (*F*_1,94_ = 0.73, *p* > 0.05). The animals pretreated with cocaine exhibited greater locomotor activity compared with animals pretreated with saline at both ages, suggesting a contextual conditioning effect. Furthermore, adolescent mice displayed higher levels of activation than adults, regardless of treatment. 

Twelve days after the last cocaine injection on Day 20 (Figure 1C), the mice were challenged with an injection of either cocaine or saline. During the abstinence phase, the mice remained in their home cages and were not subjected to handling and did not receive injections. The three-way ANOVA (age × pretreatment × challenge injection) revealed an effect of age (*F*_1,90_ = 4.39, *p* < 0.05) and an age × challenge injection interaction (*F*_1,90_ = 5.81, *p* < 0.05). The *post hoc* analysis of the interaction between age and challenge injection showed no differences between mice pretreated with saline during adolescence or adulthood. Nevertheless, the cocaine challenge injection induced greater locomotor activity in mice pretreated with cocaine during adolescence than in cocaine-treated adults. The three-way ANOVA also revealed significant main effects of pretreatment (*F*_1,90_ = 7.79, *p* < 0.01) and challenge injection (*F*_1,90_ = 59.6, *p* < 0.01) and a pretreatment × challenge injection interaction (*F*_1,90_ = 4.36, *p* < 0.05). The *post hoc* analysis confirmed that the animals treated with acute cocaine exhibited greater locomotor activity than saline-treated animals, regardless of age. Furthermore, repeated cocaine injections produced greater increases in locomotor activity than the acute injection, suggesting the expression of locomotor sensitization in both age groups.

### Anxiety-like behavior

**Figure 2:**
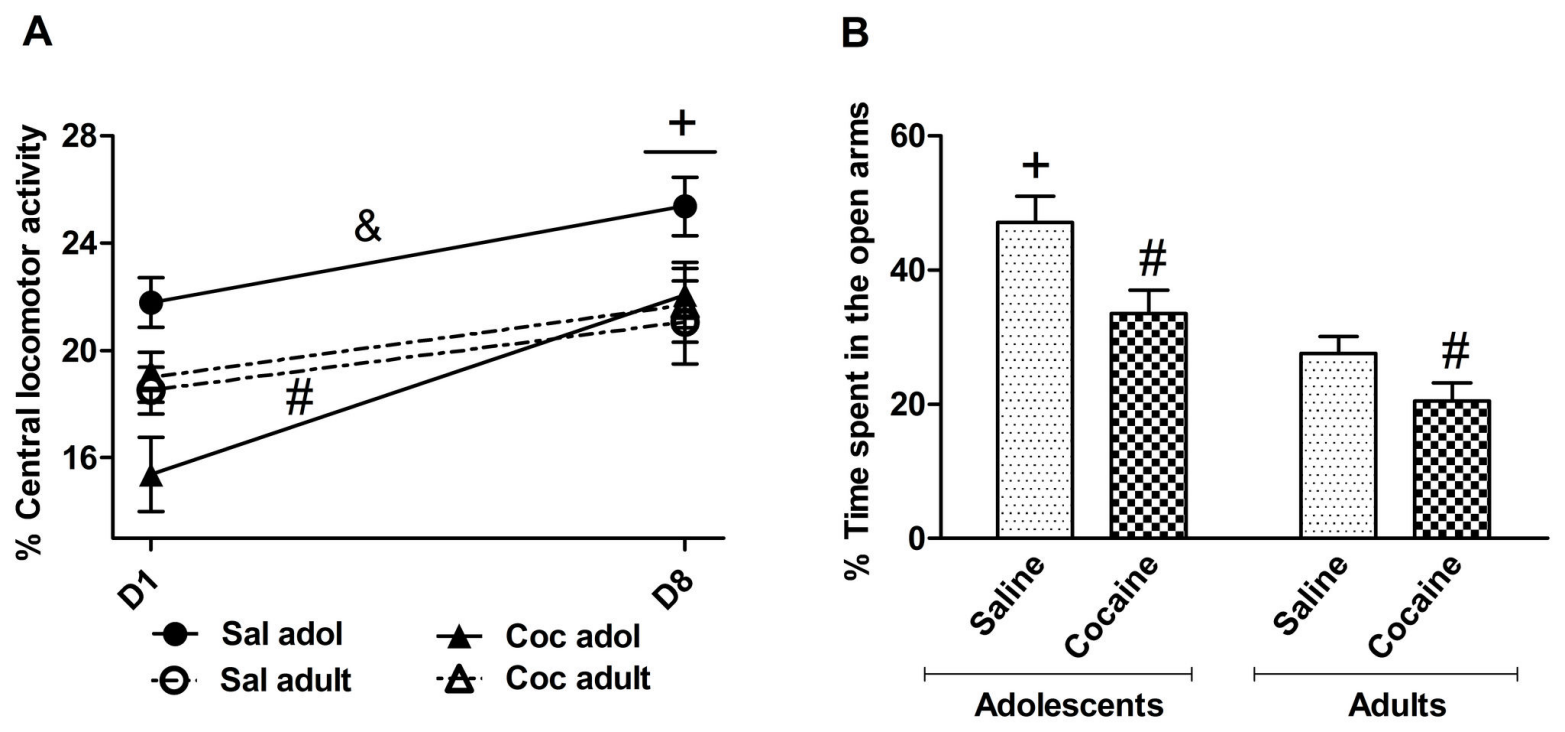
Anxiety-like behavior. (**A**) Central locomotor activity after saline or cocaine injections. Central locomotion was higher on D8 than on D1 in all of the groups (^+^ *p* < 0.01). Cocaine-treated adolescent mice exhibited lower central locomotion than their saline-treated counterparts (^#^ *p* < 0.01). Saline-treated adolescent mice exhibited greater central locomotor activity compared with all of the other groups (^&^ *p* < 0.05, *vs*. cocaine adolescent group and saline and cocaine adult groups). (**B**) Time spent in the open arms of the elevated plus maze on Day 18. Saline-pretreated adolescent mice spent more time in the open arms compared with all of the other groups (^+^ *p* < 0.05, *vs*. cocaine adolescent group and saline and cocaine adult groups). Cocaine-pretreated adolescent and adult mice spent less time in the open arms compared with their respective saline-pretreated control groups (^#^ *p* < 0.05, *vs*. saline; *n* = 25, saline adolescent [Sal adol]; *n* = 25, cocaine adolescent [Coc adol], acute cocaine; *n* = 23, saline adult [Sal adult]; *n* = 25, cocaine adult [Coc adult]). The data are expressed as mean ± SEM.

Figure 2A shows the percentage of total locomotor activity in the central region of the open field on the first and last days of repeated saline or cocaine treatment (D1 and D8). The three-way repeated-measures ANOVA (age × pretreatment × day) of the data obtained on D1 and D8 revealed significant main effects of day (*F*_1,94_ = 32.04, *p* < 0.01) and pretreatment (*F*_1,94_ = 4.85, *p* < 0.05) and an age × pretreatment interaction (*F*_1,94_ = 7.71, *p* < 0.01). An increase in central locomotor activity was observed on D8 compared with D1 in both age groups. The *post hoc* analysis of the age × pretreatment interaction showed that cocaine-treated adolescents but not cocaine-treated adults displayed lower central locomotor activity compared with the saline group, suggesting anxiety-like behavior in this age group. Moreover, saline-treated adolescent mice exhibited higher levels of central locomotion than all of the other groups, suggesting that adolescents exhibited low baseline anxiety levels.

Ten days after the last cocaine injection on Day 18, the mice were evaluated in the EPM (Figure 2B). The two-way ANOVA (age × pretreatment) revealed significant effects of age (*F*_1,94_ = 12.50, *p* < 0.01) and pretreatment (*F*_1,94_ = 27.89, *p* < 0.01) and an age × pretreatment interaction (*F*_1,94_ = 5.58, *p* < 0.05). The *post hoc* analysis of the pretreatment effect showed that cocaine-pretreated mice spent less time in the open arms than saline-pretreated mice, indicating anxiety-like behavior. The *post hoc* analysis of the interaction indicated that young mice pretreated with saline spent more time in the open arms compared with all of the other groups, indicating a reduction of the time spent in the open arms in mice pretreated with cocaine during adolescence. These results indicate the emergence of an anxiogenic-like behavioral phenotype.

### Western blot

The protein levels in each group are expressed as a percentage of the respective control group. The data are shown relative to controls and not as absolute values. The CREB and pCREB bands were visualized at approximately 43 kDa, and α-tubulin was visualized at approximately 50 kDa.

**Figure 3:**
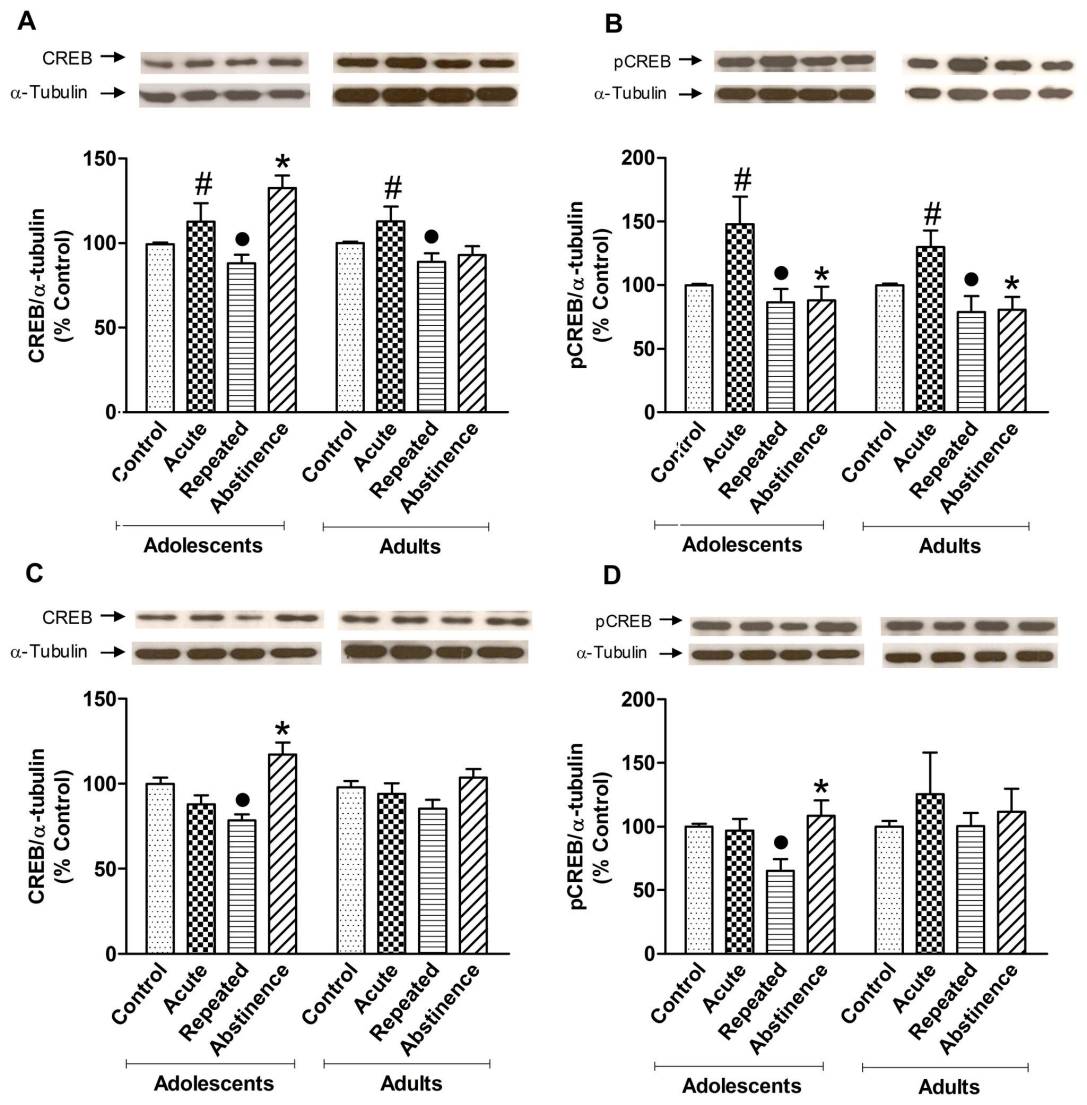
CREB and pCREB Western blot. (**A**) CREB protein levels in the PFC. Acute cocaine increased CREB levels (^#^ *p* < 0.05, vs. control group; pretreatment × challenge interaction), whereas repeated cocaine decreased CREB levels (^●^ *p* < 0.05, vs. acute group). Abstinence in mice pretreated with cocaine during adolescence increased CREB levels compared with all of the other groups (*p < 0.05, *vs*. all groups, considering pretreatment and age). (**B**) pCREB protein levels in the PFC. The acute cocaine groups exhibited higher pCREB levels compared with their respective control groups (^#^ *p* < 0.05, *vs*. control), whereas the repeated and abstinence groups displayed lower pCREB levels compared with their respective acute groups, both in adolescent and adult mice (^●^ *p* < 0.05, repeated *vs*. acute and abstinence *vs*. acute). (**C**) CREB protein levels in the hippocampus. In adolescents, repeated cocaine decreased CREB levels compared with the control group (^●^ *p* < 0.01, *vs*. control group). Abstinence increased CREB protein levels compared with the saline and repeated groups (*p < 0.01). (**D**) pCREB protein levels in the hippocampus. Repeated cocaine decreased pCREB levels compared with the acute and abstinence groups (^●^ *p* < 0.05). pCREB levels in the abstinence group were higher than in the repeated group (*p < 0.05). Adolescent groups: control (*n* = 7 for all groups, except for the pCREB-Hippocampus, which was *n* = 6), acute cocaine (*n* = 7 for all groups), abstinence (*n* = 7 for all groups), repeated cocaine (*n* = 8 for all groups, except for CREB and pCREB in the hippocampus, which was *n* = 7). Adult groups: control (*n* = 7 for all groups), acute cocaine (*n* = 7 for all groups), abstinence (*n* = 7 for all groups), repeated cocaine (*n* = 8 for all groups, except for pCREB in the PFC, which was *n* = 7). The data are expressed as mean ± SEM.

Figure 3 shows the protein levels of CREB (Figure 3A) and pCREB (Figure 3B) in the PFC in young and adult mice and a representative Western blot gel. The CREB and pCREB results were first analyzed using a three-way ANOVA (age × pretreatment × challenge injection). When necessary, a two-way ANOVA (pretreatment × challenge injection) was performed for each age group. The three-way ANOVA of CREB in the PFC revealed an effect of age (*F*_1,50_ = 5.01, *p* < 0.05), a pretreatment × challenge injection interaction (*F*_1,50_ = 20.14, *p* < 0.01), a pretreatment × age interaction (*F*_1,50_ = 5.30, *p* < 0.05), an age × challenge injection interaction (*F*_1,50_ = 5.86, *p* < 0.05), and an age × pretreatment × challenge injection interaction (*F*_1,50_ = 6.13, *p* < 0.05).

The *post hoc* analysis of the pretreatment × challenge injection interaction revealed an elevation of CREB levels after acute cocaine and a reduction of CREB levels after repeated cocaine administration compared with the acute group. After abstinence, only the mice treated with cocaine during adolescence exhibited higher CREB levels compared with all of the other groups.

The three-way ANOVA of pCREB in the PFC revealed effects of pretreatment (*F*_1,49_ = 21.67, *p* < 0.01) and challenge injection (*F*_1,49_ = 5.74, *p* < 0.05) and a pretreatment × challenge injection interaction (*F*_1,49_ = 7.19, *p* < 0.01). No significant effect of age was detected (*F*_1,49_ = 1.08, *p* > 0.05). Thus, separate two-way ANOVAs (pretreatment × challenge injection) were conducted for each age group. In the adolescent group, we observed a main effect of pretreatment (*F*_1,25_ = 8.68, *p* < 0.01) and a pretreatment × challenge injection interaction (*F*_1,25_ = 3.91, *p* < 0.05). The *post hoc* analysis revealed an increase in pCREB levels after acute cocaine administration compared with the control group, whereas repeated cocaine administration and abstinence decreased pCREB levels compared with the acute group. In the adult group, we detected a main effect of pretreatment (*F*_1,25_ = 21.03, *p* < 0.01) and a pretreatment × challenge injection interaction (*F*_1,25_ = 5.48, *p* < 0.05). The *post hoc* analysis revealed an increase in pCREB levels after acute cocaine administration compared with the control group, whereas repeated cocaine administration and abstinence decreased pCREB levels compared with the acute group.

Figure 3C shows the protein levels of CREB, and Figure 3D shows the protein levels of pCREB in the hippocampus in young and adult mice, including a representative Western blot gel. The three-way ANOVA of CREB levels in the hippocampus revealed an effect of challenge injection (*F*_1,50_ = 25.3, *p* < 0.01) and a pretreatment × challenge injection interaction (*F*_1,50_ = 7.47, *p* < 0.01). No significant effect of age was detected (*F*_1,50_ = 25.32, *p* > 0.05). Thus, separate two-way ANOVAs (pretreatment × challenge injection) were conducted for each age. In the adolescent group, we detected a main effect of challenge injection (*F*_1,25_ = 25.44, *p* < 0.01) and a pretreatment × challenge injection interaction (*F*_1,25_ = 6.06, *p* < 0.05). The *post hoc* analysis revealed a decrease in CREB levels after repeated cocaine administration compared with the control group, whereas abstinence increased CREB levels compared with the control and repeated cocaine administration groups. In adults, the two-way ANOVA did not detect significant differences (*F*_1,25_ = 2.06, *p* > 0.05).

The three-way ANOVA of pCREB levels in the hippocampus revealed a pretreatment × challenge injection interaction (*F*_1,49_ = 3.93, *p* > 0.05). Separate two-way ANOVAs (pretreatment × challenge injection) were performed for each age group. In adolescent mice, the two-way ANOVA revealed an effect of challenge injection (*F*_1,24_ = 5.66, *p* < 0.05) and an interaction (*F*_1,24_ = 4.2, *p* < 0.05). Lower pCREB levels were detected in mice repeatedly treated with cocaine compared with the acute and abstinence groups. No significant differences were found in adult mice (*F*_1,25_ = 0.96, *p* > 0.05).

Table 2 shows the pCREB/CREB ratio in the PFC and hippocampus.

**Table 2:** Western blot analysis of pCREB/CREB ratio.

**Age**	**Brain region**	**Control**	**Acute**	**Repeated**	**Abstinence**
**Adolescent**	**PFC**	**1.005 ± 0.004**	**1.314 ± 0.168**	**1.011 ± 0.115**	0.683 ± 0.090* ^+^
**Adult**	**PFC**	**1.001 ± 0.017**	**1.161 ± 0.079**	**0.892 ± 0.109**	**0.880 ± 0.083**
**Adolescent**	**Hippocampus**	**1.007 ± 0.042**	**1.100 ± 0.044**	**0.840 ± 0.097** ^+^	**1.019 ± 0.048**
**Adult**	**Hippocampus**	**1.027 ± 0.044**	**1.342 ± 0.328**	**1.201 ± 0.106**	**1.090 ± 0.189**

pCREB/CREB ratio values in the PFC and hippocampus in mice pretreated with saline or cocaine during adolescence or adulthood and challenged with saline (control and abstinence groups) or cocaine (acute and repeated groups). The experimental design is described in Table 1. The data are expressed as mean ± SEM. *p < 0.05, compared with control group; ^+^ *p* < 0.05, compared with the acute group.

The three-way ANOVA of the pCREB/CREB ratio in the PFC revealed effects of pretreatment (*F*_1,49_ = 13.46, *p* < 0.01) and challenge injection (*F*_1,49_ = 8.55, *p* < 0.01) but no effect of age (*F*_1,49_ = 0.8, *p* > 0.05) and no interaction. The deconstruction of the data in the one-way ANOVAs (control × acute × repeated × abstinence) for each age group revealed that abstinence from repeated cocaine in the adolescent group decreased the pCREB/CREB ratio in the PFC compared with the control and acute groups (*F*_3,25_ = 5.14, *p* < 0.01). No difference was found in the adult group (*F*_3,24_ = 2.65, *p* = 0.07).

The three-way ANOVA of the pCREB/CREB ratio in the hippocampus revealed no significant differences. Individual one-way ANOVAs performed for each age group revealed that repeated cocaine in the adolescent group decreased the pCREB/CREB ratio in the PFC compared with the acute group (*F*_3,24_ = 3.02, *p* < 0.05). No difference was found in the adult group (*F*_3,25_ = 0.49, *p* = 0.68).

### Correlations between sensitization and CREB and pCREB levels

**Figure 4:**
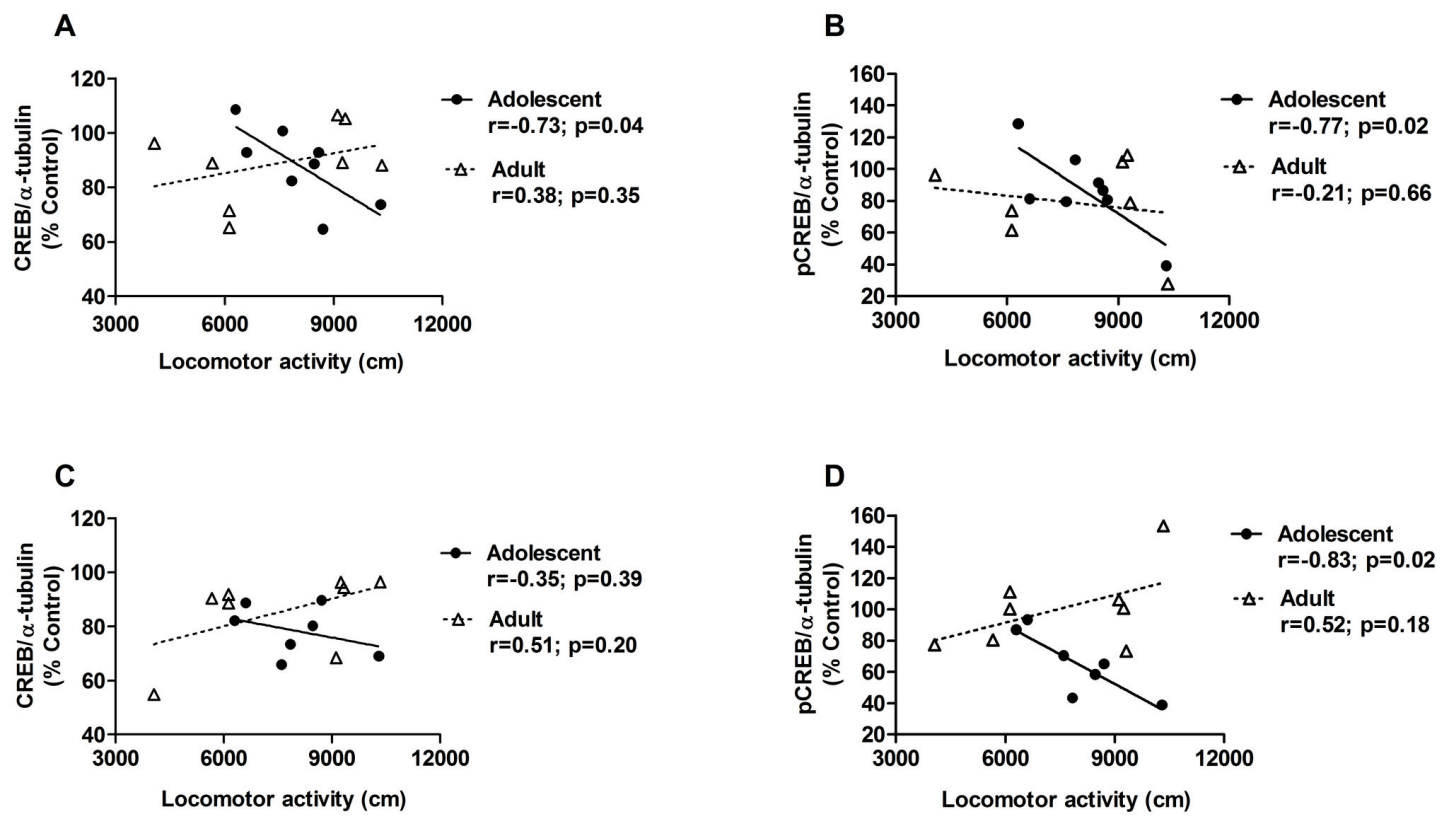
Correlations between sensitization and CREB and pCREB levels. A significant negative correlation was observed between cocaine-induced sensitization and (**A**) CREB levels and (**B**) pCREB levels in the PFC and (**C**) pCREB in the hippocampus in adolescent mice. No relationship was observed between sensitization and CREB or pCREB levels in adult mice.

To assess the possible relationships between locomotor sensitization and CREB levels, correlation analyses were performed (Figure 4). The Pearson test revealed significant negative correlations between locomotor activity and CREB levels (*r* = -0.7291, *p* = 0.04) and between locomotor activity and pCREB levels (*r* = -0.7729, *p* = 0.02) in the PFC in adolescent mice, whereas no significant correlation was detected in adult mice for CREB (*r* = 0.38, *p* = 0.35) or pCREB (*r* = -0.2062, *p* = 0.66). These results indicate that adolescents with greater locomotor activity (i.e., cocaine-induced sensitization) exhibited lower CREB levels in the PFC. A significant correlation was found between locomotor activity and pCREB levels in adolescent mice (*r* = -0.833, *p* = 0.02) but not in adult mice (*r* = 0.52, *p* = 0.18) in the hippocampus. No correlations were found between locomotor activity and CREB levels in adolescent mice (*r* = -0.35, *p* = 0.39) or adult mice (*r* = 0.51, *p* = 0.20) in the hippocampus.

## Discussion

The present study confirmed previous data from our laboratory, in which mice pretreated with cocaine during adolescence displayed a more robust expression of sensitization to cocaine compared with their adult counterparts [27]. We investigated the behavioral and neurochemical consequences of cocaine withdrawal (abstinence) in mice sensitized to cocaine during adolescence and compared them to mice sensitized during adulthood. We also observed an anxiogenic-like phenotype in cocaine-abstinent adolescents compared with their own control group. The observed differences in behavioral sensitization between adolescent and adult mice may represent a critical time period in the ontogenic risk for drug dependence.

A contextual conditioning effect was also observed when the animals were tested in the open field after a saline injection, reflected by increased locomotion in cocaine-pretreated mice compared with saline-pretreated mice. This result is consistent with several previous studies that demonstrated the involvement of a strong context-dependent component in behavioral sensitization [12], although it also occurs when the drug is administered with minimal exposure to the test environment [30].

In the present study, adolescents exhibited a greater magnitude of behavioral sensitization compared with adults. The attribution of incentive salience (i.e., “drug wanting”) of a drug has been linked to the neurotransmitter dopamine [31]. Behavioral sensitization is associated with the incentive salience of a drug, and cocaine “wanting” is likely more evident in adolescents than in adults. Epidemiological evidence indicates that the earlier initiation of substance use in youths is associated with a higher risk of developing substance abuse and dependence [1]. Moreover, Robinson and Berridge [12] hypothesized a link between behavioral sensitization and relapse. Bartlett et al. [32] reported a trend toward a greater number of rehospitalizations, indicative of relapse, among patients who developed cocaine-induced paranoia sensitization. As a result, we hypothesize that adolescents have a greater risk of relapsing following cocaine abstinence because of the heightened sensitization properties of cocaine in adolescents *vs.* adults. Indeed, adolescent rats exhibited delayed extinction, greater reinstatement of cocaine-induced conditioned place preference [2], and faster cocaine-induced dopamine peaks in the nucleus accumbens compared with adults [27,33].

Some studies have suggested that neurochemical alterations that contribute to differences in the behavioral responses to cocaine between adults and adolescents are mediated primarily by the differential degree of maturation in dopaminergic brain systems [34-36]. As a result, the early increase in dopamine release in adolescent mice *vs.* adults may have driven the enhancement of sensitization observed in adolescent *vs.* adult mice [37]. One possibility is that cocaine accelerates the development of the dopaminergic systems and dopaminergic functions that underlie behavioral sensitization.

 We also assessed the anxiety-like effects of cocaine in adolescent and adult mice in two different phases of treatment (i.e., during cocaine withdrawal and during cocaine administration) using the EPM and evaluating thigmotaxis in the open field test, respectively. Although the adolescent period has been considered particularly sensitive to stressful conditions [10], saline-treated adolescent mice exhibited a higher expression of thigmotaxis (from Day 1 to Day 8 of treatment) and showed an increase in the percentage of entries into the open arms of the EPM and spent more time in the open arms on Day 18 compared with saline-treated adult mice. Even when considering the temporal gap, the data appear to be consistent. This suggests lower basal anxiety-like behavior in adolescents. During repeated treatment (Day 1 to Day 8), cocaine significantly decreased central locomotor activity in adolescents but not in adults.

 During cocaine abstinence, the animals preexposed to cocaine exhibited higher levels of anxiety-like behavior than saline-treated mice, regardless of age. However, young mice showed more cocaine abstinence-induced anxiety-like behavior compared with their respective saline controls. This difference was not detected in adult mice. Although the baseline levels differed between young and adult mice, the data suggest that adolescent mice are more sensitive than adults to cocaine-induced anxiety. This may indicate behavioral perturbation related to substance abuse disorders. The anxiogenic effects of cocaine withdrawal have been observed after both short and long withdrawal periods in adult rats [38] but not in adult mice [39], although Costall et al. [40] reported an anxiogenic response to cocaine withdrawal in mice.

 High comorbidity exists between anxiety and drug addiction. High anxiety is one of the risk factors that predisposes individuals to drug addiction [41] and may contribute to greater relapse rates. For example, a high index of anxiety, assessed by self-grooming behavior, has been associated with enhanced motivation to self-administer cocaine [42]. Anxiety may also increase the rewarding effects of cocaine, reflected by the enhancement of place conditioning induced by cocaine in rats [43]. Consistent with our data, evidence suggests that the exacerbation of anxiety induced by cocaine withdrawal is accompanied by dysfunctions in the dorsal mPFC [44].

 To address the possible role of CREB in cocaine sensitization, we tested whether CREB and pCREB are differentially regulated in the PFC and hippocampus in animals treated with saline or cocaine during adolescence or adulthood.

Acute cocaine administration induced CREB and pCREB activation in the PFC, and both adolescent and adult mice developed tolerance to this effect following repeated cocaine administration. Conversely, cocaine abstinence substantially increased CREB levels in the PFC and hippocampus and pCREB in the hippocampus compared with repeated administration in adolescent mice but not in adult mice. This demonstrates the presence of a homeostatic neuroadaptive response to the reduction of the levels of these transcription factors following repeated cocaine administration. This homeostatic regulation of CREB and pCREB was not observed in adult mice, suggesting that adolescent mice are more susceptible to neuroplastic changes induced by cocaine and cocaine withdrawal in the PFC and hippocampus.

The analysis of the pCREB/CREB ratio revealed that abstinence from repeated cocaine decreased pCREB levels and the pCREB/CREB ratio in the PFC. However, this decrease in the pCREB/CREB ratio appeared to be attributable to an increase in CREB levels and not to a decrease in pCREB levels. CREB levels did not remain stable following cocaine abstinence. Thus, the present results suggest that analyzing total CREB levels and pCREB levels separately is important for the interpretation of age-dependent changes in transcription factors. In the PFC, CREB levels were dynamically altered in cocaine-abstinent adolescent mice, and we found age-related changes upstream of CREB activation.

Edwards et al. [20] showed that cocaine produced similar increases in CREB phosphorylation following acute and chronic cocaine administration in the PFC in adult rats. In the present study, we observed a neuroadaptive response after repeated cocaine injections in both adolescent and adult mice. This difference may be attributable to species differences (rats vs. mice), although our finding is consistent with their study, in which no alterations in pCREB were found in the hippocampus in adult rodents following acute or chronic cocaine administration.

Evidence suggests that this specific CREB activation in abstinent adolescent mice may be responsible for facilitating Pavlovian associative learning promoted by drug-induced plasticity. Dopamine D_1_-type receptor activity in the PFC can influence the storage of long-term memory [45,46]. A high dose of a selective dopamine D_1_-type receptor agonist in rats facilitated the recovery of information in recognition memory tests of the position of objects and temporary memory after a period of 4 h but not after 15 min [47]. This D_1_-dependent improvement in memory has been associated with an increase in the phosphorylation of CREB and DARPP-32 in the PFC [48]. Similarly, the activation of CREB-dependent gene expression in the hippocampus by monoaminergic or dopaminergic signaling facilitates the induction of long-term potentiation (LTP) and LTP-dependent learning processes during arousal and attention [49]. This modulatory effect could lead to a favorable learning state, in which a relatively lasting change in neuronal excitability would support facilitation in learning tasks [50]. Although speculative, the increased CREB levels found in the PFC and hippocampus in cocaine-withdrawn adolescents in the present study could be involved in the facilitation of Pavlovian association promoted by drug-induced plasticity. Supporting this possibility, the activation of CREB in the PFC and hippocampus in rodents has been shown to be related to an increase in cocaine-induced conditioned place preference [51,52].

Decreases in dopamine release in the nucleus accumbens have been linked to the regulation of drug- and non-drug-induced aversive affective states [53,54]. Negative emotional states have been associated with repeated CREB activation, which in turn promotes dynorphin expression in the nucleus accumbens and decreases dopaminergic activity [55]. These negative emotional symptoms of cocaine withdrawal may serve as a motivational trigger to readminister the drug to alleviate the aversive aspects of abstinence [56]. In fact, the abrupt cessation of drug abuse can cause physical and emotional withdrawal symptoms [55] that are considered allostatic adjustments that occur in response to drug-induced neural changes, usually opposite to the effects of the drug. The overexpression of CREB in the nucleus accumbens decreases the rewarding effects of drugs and increases the aversive effects of cocaine withdrawal [56] and cocaine-primed reinstatement [57]. These processes are known to contribute to the unpleasant symptoms that result from repeated use in humans [32] that ultimately contributes to the maintenance of drug consumption. Although the nucleus accumbens has been the focus of most studies on the neurobiology of behavioral sensitization, reinstatement, and reward, the participation of other brain regions, such as the PFC and hippocampus, is clearly evident [31]. Although no proof of causality of exists, increased CREB in abstinent adolescents may contribute to increased withdrawal-associated anxiety.

The importance of PFC dopamine neurotransmission in cocaine-induced behavioral sensitization has been well documented [58]. Behavioral sensitization to cocaine is associated with a blunted D_2_ receptor-dependent modulation of action-potential firing in PFC interneurons, which could contribute to functional hypoactivation of the PFC [59]. Abstinent cocaine abusers exhibit impaired function of the lateral PFC in top-down processes [60]. In adolescents, ongoing maturation of the PFC supposedly contributes to a greater risk of developing substance use disorders [7].

In the present study, we also demonstrated a negative correlation between locomotor activity and CREB and pCREB levels in the PFC and pCREB in the hippocampus in adolescent mice. These correlations support the hypothesis of an association between cocaine-induced decreases in CREB and behavioral sensitization in adolescent but not adult mice. This is particularly interesting because mice pretreated with cocaine during adolescence displayed greater expression of behavioral sensitization than adults.

 In summary, the present study found that repeated cocaine administration and withdrawal induced distinct behavioral and neurochemical effects that differed between adolescent and adult mice. Adolescent mice were more susceptible to some of the neuroplastic changes induced by withdrawal from repeated cocaine administration in the PFC and hippocampus compared with adult mice, suggesting that the action of psychoactive substances in a still-developing nervous system can have more profound effects than in an already mature nervous system. Differential alterations in CREB function in the PFC and hippocampus may have important implications for the higher susceptibility to cocaine addiction during adolescence or later in life.
